# Nanodrugs with intrinsic radioprotective exertion: Turning the double‐edged sword into a single‐edged knife

**DOI:** 10.1002/EXP.20220119

**Published:** 2023-03-31

**Authors:** Jiaming Guo, Zhemeng Zhao, Zeng‐Fu Shang, Zhongmin Tang, Huanhuan Zhu, Kun Zhang

**Affiliations:** ^1^ Department of Radiation Medicine, College of Naval Medicine Naval Medical University Shanghai China; ^2^ National Engineering Research Center for Marine Aquaculture, Marine Science and Technology College Zhejiang Ocean University Zhoushan China; ^3^ Department of Radiation Oncology Simmons Comprehensive Cancer Center at UT Southwestern Medical Center Dallas Texas USA; ^4^ Department of Radiology University of Wisconsin‐Madison Madison Wisconsin USA; ^5^ Central Laboratory, Shanghai Tenth People's Hospital Tongji University School of Medicine Shanghai P. R. China; ^6^ National Center for International Research of Bio‐targeting Theranostics Guangxi Medical University Nanning Guangxi P. R. China; ^7^ Department of Oncology, Sichuan Provincial People's Hospital, School of Medicine University of Electronic Science and Technology of China Chengdu Sichuan P. R. China

**Keywords:** biosafety, intrinsic radioprotection, nanomaterials

## Abstract

Ionizing radiation (IR) poses a growing threat to human health, and thus ideal radioprotectors with high efficacy and low toxicity still receive widespread attention in radiation medicine. Despite significant progress made in conventional radioprotectants, high toxicity, and low bioavailability still discourage their application. Fortunately, the rapidly evolving nanomaterial technology furnishes reliable tools to address these bottlenecks, opening up the cutting‐edge nano‐radioprotective medicine, among which the intrinsic nano‐radioprotectants characterized by high efficacy, low toxicity, and prolonged blood retention duration, represent the most extensively studied class in this area. Herein, we made the systematic review on this topic, and discussed more specific types of radioprotective nanomaterials and more general clusters of the extensive nano‐radioprotectants. In this review, we mainly focused on the development, design innovations, applications, challenges, and prospects of the intrinsic antiradiation nanomedicines, and presented a comprehensive overview, in‐depth analysis as well as an updated understanding of the latest advances in this topic. We hope that this review will promote the interdisciplinarity across radiation medicine and nanotechnology and stimulate further valuable studies in this promising field.

## Graphical abstract

The realization of ideal radioprotectors has long been the hotspot in radiation medicine. Recently, nano‐technology is equipping the traditional radioprotectors with better bioavailability, less toxicity, etc. This review summarized the development, design innovations, applications, challenges, and prospects of the intrinsic antiradiation nanomedicines, and presented a comprehensive overview, in‐depth analysis, and an updated understanding of the latest advances in this topic.

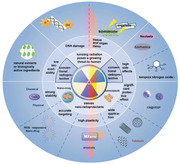

## 1 INTRODUCTION

Like a double‐edged sword, the fast‐developing nuclear technology confers ionizing radiation (IR) both increasingly benefits and potential harms to people. For example, in modern human medicine, IR plays a more and more important role due to its rising extensive exploration of radiotherapy in various refractory cancers. Currently, most cancer patients require radiation therapy alone or in combination with other treatments, such as surgery, chemotherapy, and immunotherapy.^[^ ^1^, ^2^, ^3^ ^]^ As one of the conventional cancer remedies, radiotherapy has been widely used in clinical practices thanks to the tumor cell‐killing effect of ionizing, which, nevertheless, also inevitably causes damages to surrounding healthy cells or tissues in a dose‐dependent manner, consequently compromising its therapeutic effect to some extent. In addition, in some other scenarios including nuclear bomb explosions, nuclear accidents, dirty bomb attacks, and so on, IR poses a serious threat to people. Hence, the elimination of IR health‐related injuries is key to its transformation into a single‐edged knife that remains purely beneficial. As a result, IR protection has long been a hot research topic that is of paramount importance to safeguard against nuclear emergencies and at the same time improves the efficacy of radiotherapy against tumors, which is of great importance and very worthy of further research.

It is widely accepted that IR may cause damages to biological tissues in both direct and indirect ways. IR, represented by α particles, β particles, γ‐rays, X‐rays, etc., is a kind of high‐energy radiation that can exert the ionization of matter, during which the intermediate ions and free radicals destroy the chemical structure of DNA and other biomolecules and are highlighted since they can pose a serious hazard to the organism and endanger human health. When IR‐induced DNA damage exceeds the self‐repair ability of cells, incorrect DNA repair leads to chromosome aberrance or even cell death, which is usually lethal to the organism.^[^ ^4^, ^5^, ^6^ ^]^ On one hand, IR penetrates the body and deposits energy directly at the biological target molecules, directly leading to destructive effects on molecule structure associated with the breakages of chemical bonds including DNA, lipids, proteins, etc. On the other hand, IR first interacts with materials (commonly such as the surrounding water within the body) around the target biomolecules, and then produces a series of reactive oxygen species (ROS) such as superoxide •O_2_^−^, hydrogen peroxide (H_2_O_2_), and hydroxyl radicals (•OH). Immediately afterward, the IR‐induced ROS further destroys the biological molecules and cellular structure, leading to indirect destructive effects, eventually resulting in cell dysfunction, catastrophe, and even death.^[^ ^7^, ^8^, ^9^ ^]^ Excessive ROS is preferable to kill tumor cells and repress cancer progression via direct apoptosis and activated immune responses,^[^ ^10^, ^11^, ^12^, ^13^, ^14^, ^15^, ^16^, ^17^ ^]^ but for normal tissues or organs, it is a hazard.^[^ ^18^ ^]^ With arising from the primary damages mentioned above, a variety of radiation diseases sprouts gradually. Typically, many complications induced by radiotherapy, including acute radiation syndrome (ARS), radiation‐induced lung injury (RILI),^[^ ^19^, ^20^ ^]^ radiation skin injury,^[^ ^21^ ^]^ radiation myocardial injury,^[^ ^22^ ^]^ radiation pelvic inflammatory, radiation injury of the intestine (RII),^[^ ^23^ ^]^ etc., are often accompanied in clinical radiotherapy practice, and their severity associates with the irradiated site, dose, and dose rate and other factors. For example, the treatment site is usually located on the head and neck area when radiotherapy is applied to cure brain tumors. Under such circumstances, the salivary glands and the covered mucous membrane on the head and neck are accessible to suffer from radiation injuries.^[^ ^24^ ^]^ In addition, if one comes across the radiotherapy adverse complications, he/she would show symptoms such as mucositis, esophagitis, xerostomia, and gastrointestinal bleeding that are unfavorable to the prognosis of patients. Collectively, during combat between humans and cancer, despite radiotherapy being a powerful weapon to kill tumor cells, the protection of healthy cells or tissues is also an important concern or task that should not be ignored.^[^ ^25^ ^]^ In this context, the design and development of radioprotective drugs are of great significance and urgently demanded cancer treatment and various IR‐induced diseases.

Due to the vital role of radioprotection, many endeavors have been done to seek ideal radioprotectants (i.e., the radioprotective agents), but no perfect one has been accomplished yet due to several bottle‐necked reasons. However, the rapid development of nanotechnology provides new powerful tools to handle these challenges. Amounts of excellent researches focused on the innovation of nano radioprotective drugs have been carried out and reported, which has been reviewed elsewhere from various other perspectives.^[^ ^26^, ^27^, ^28^, ^29^, ^30^, ^31^ ^]^ On the one hand, some of those reviews concentrated on more specific types of nanomaterials applied in radioprotection. For example, Anita Krokosz et al. summarized the carbon nanoparticles with the potential to be radioprotectors in 2016;^[^ ^30^ ^]^ Xiuling He et al. discussed ROS‐scavenging nanomaterials in 2021;^[^ ^31^ ^]^ and Ruiying Zhao et al. and Yu Chong et al. focused on the catalytic nanoenzymes in 2021 and 2022, respectively.^[^ ^26^, ^28^ ^]^ It is worth noting that the topics of the above‐mentioned reviews all belong to the intrinsic nano‐radioprotectant class discussed in our current paper. Compared to them, we first systemically discuss the recent development of the intrinsic nano‐radioprotectants on a broader scale containing the nanosized, enzyme‐mimicking, stimuli‐responsive, combined strategy‐based, and other kinds of intrinsic nano‐radioprotectants. On the other hand, Jiani Xie et al. published another review that encompassed a large range of nanomaterials related to radioprotection in 2018.^[^ ^29^ ^]^ They mainly described two groups of nanomaterials (named as multifunctional nanomaterials) with radioprotective ability: radioprotectors delivered by nanocarriers and other nano radioprotectors. In comparison, we mainly focused on the intrinsic reagents applied in radiation protection (the most important and extensively studied class in this area) and intensively elaborated the brief developmental history, design principles, bio‐applications, limitations, and future advice of the intrinsic antiradiation nanomedicines in detailed subclasses with mostly referring the latest studies. Thus, it is both an update for this research domain and a comprehensive and in‐depth review that can interest and inspire readers with either radiation protection or nanomaterial background.

Considering all the above, the current review is fabricated elaborately to present a comprehensive perspective that is both broad and deep. To begin, we relook at the development and dilemma of radioprotective drug exploitation. Then, we summarize the advantages and design strategies of the intrinsic nano‐radioprotectants. After that, the research status of intrinsic nano drugs for radioprotection is highlighted in detail. Finally, the existing challenges and suggestions for future studies in this field are discussed. Hopefully, the present review will be thought‐provoking for researchers who wish to learn about the latest advancements in intrinsic radioprotectants and stimulate further research in this exciting field.

## 2 RESEARCH AND DEVELOPMENT HISTORY, CURRENT SITUATION, AND BOTTLENECKS OF RADIOPROTECTIVE DRUGS

**FIGURE 1:**
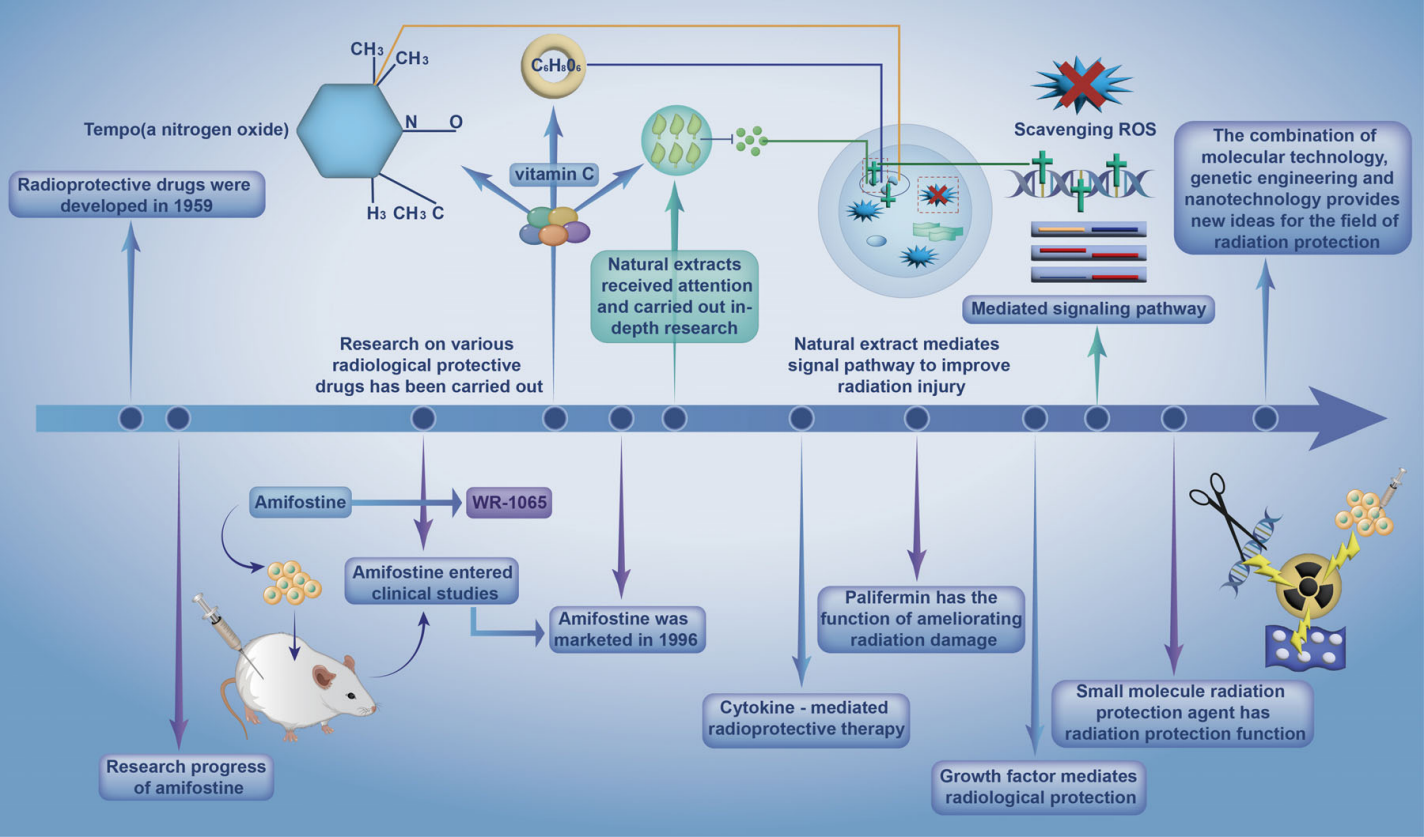
A brief overview of the advancement of conventional radioprotective drugs, wherein the cutting‐edge progress of radiation protection drugs such as amine, vitamin C, natural extracts, growth factor, and small molecule are summarized.

Radiation therapy relies heavily on the research and application of radioprotective chemical agents. Many different radioprotective compounds have been developed to prevent and/or cure IR damages. To achieve the goal of radioprotection, various mechanisms corresponding to their certain radiopharmaceuticals have been adopted, including direct free radicals scavenging, DNA repair enhancement, misaligned cell cycle rectification, growth factor and cytokines exudation regulations, antioxidant capability improvement, IR‐induced apoptosis inhibition, hematopoietic cell regeneration promotion after introducing stem cell therapy, etc.^[^ ^32^ ^]^ Among them, the most common strategy is to explore new free radical scavengers, while the most effective approach is to optimize the immune functions via controlling cytokines and redox genes. In this regard, gene and stem cell therapies have also gained much attention in recent years (Figure 1).^[^ ^33^ ^]^

As a classical radioprotectant, amifostine has always played an important role in the field of radiation protection. As early as 1959, the US Army Anti‐Radiation Drug Development Program of the Walter Reed Institute of Research began to screen potential radioprotective chemicals. Their researchers screened out a new synthetic aminothiol with the best radiation protection efficacy and the lowest toxicity out of over 4,400 sulfhydryl‐containing compounds that were proved to protect cells from radiation. This is the world‐famous amifostine (identification number: WR‐2721) that is also known as 2‐(3‐aminopropyl) aminoethyl thiophosphate. Since then, studies regarding amifostine including in vitro dish studies and in vivo animal experiments have proceeded successively. Amifostine entered human clinical trials in the 1970s and 1980s and performed well. In 1996, the U.S. Food and Drug Administration (FDA)‐approved it as the first clinical radioprotective drug, and it has since been a key player in the radiation protection area.^[^ ^34^, ^35^, ^36^ ^]^ Moreover, many kinds of new molecular compounds with the ability to attenuate toxic free radicals have always been selected as potential candidates for radiation injury prevention. One of the most famous representatives is the molecular hydrogen (hydrogen gas, H_2_). In our previous work, we first found that H_2_ could exhibit broad‐spectrum radioprotective competencies including injuries from the hematopoiesis system, cardiovascular system, reproductive system, respiratory system, immune system, etc.^[^ ^37^, ^38^, ^39^ ^]^ Afterward, many other studies made much success in the creation and exploration of potent nanomaterials with the efficient and intelligent H_2_‐releasing capability, which further improved its efficacy greatly.^[^ ^40^, ^41^, ^42^ ^]^ Recently, Qianjun He's team has revealed that Fe‐porphyrin is a H_2_‐targeted molecule, which can self‐catalyze the hydrogenation/reduction by reacting with H_2_ to catalytically scavenge ∙OH, revealing the basic mechanism under the extensive radioprotective effects of H_2_.^[^ ^43^ ^]^

Apart from the ROS‐scavenging principle, there are several other mechanisms usually adopted for developing radioprotectants. First, the materials featuring reactive nitrogen species scavenging are made full use of to design novel substances for radioprotection.^[^ ^44^ ^]^ Second, some researchers found that some cytokines or growth factors over a specific period injection in animal studies could reduce radiation damage. At present, a series of chemical drugs, such as palifermin (another FDA‐approved radioprotectant), neulasta, leukine, eltrombopag, entolimod, genistein, have been validated to serve as radioprotectants of this kind and perform well in the field of radiation protection.^[^ ^45^, ^46^, ^47^, ^48^, ^49^, ^50^, ^51^, ^52^ ^]^ Third, some vitamins that are also known as antioxidants can protect against radiation injuries by interrupting the free radicals‐induced chain reaction.

As well, some other studies emphasized the role of extracting natural products in preventing radiation damage.^[^ ^53^, ^54^, ^55^, ^56^ ^]^ Natural extracts or biologically active ingredients in plants can reduce oxidative stress‐arised damages and enhance the body's antioxidant ability, endowing them with a considerable ability to boost radiation protection.^[^ ^57^ ^]^ As a paradigm, flavonoids as a class of natural substances isolated from plants are originally extracted from Chinese herbal medicine, and they usually include epicatechin, apigenin, and silymarin, all of which have been proven to have neuroprotective and radioprotective characteristics.^[^ ^58^, ^59^, ^60^ ^]^ In principle, flavonoids as potent antioxidants can inhibit regulatory enzymes or transcription factors involved in the control of inflammatory mediators, influence oxidative stress through interactions with DNA, and enhance genomic integrity.^[^ ^61^ ^]^ On basis of this identical principle, natural substances including ethyl caffeate, curcumin, and thymol also succeed in scavenging oxidative stress and exerting radiation protection actions. The other important members of natural extracts‐derived radioprotectants are curcumin (CUR) and resveratrol (RSV). CUR is a rare diketone pigment that is ubiquitous in plants and can be isolated from the rhizomes of Zingiberaceae or Araceae plants, and it is usually used as a food colorant because of its low toxicity.^[^ ^62^ ^]^ As a natural polyphenol compound, CUR has been demonstrated to boost antioxidant enzyme activity, and reduce oxidative damages resulting from radiation therapy. Accumulative studies have shown that CUR is imparted with radioprotective functions on the hematopoietic system, skin, lungs, hearts, eyes, brain tissue, and other organs.^[^ ^63^, ^64^, ^65^, ^66^, ^67^, ^68^, ^69^, ^70^ ^]^ Impressively, an NIR‐triggered TPGS‐BCNPs has been recently constructed to load and deliver curcumin to enhance its bioavailability, which has been well proven to exhibit enhanced radioprotective capability for normal tissues. In addition, the excellent performance of RSV on radioprotection inspired the scientists as well.^[^ ^71^ ^]^ As a natural non‐flavonoid (*trans*‐3, 5, 40‐trihydroxy stilbene) polyphenol, it exists widely in red grape skin and displays a variety of biological activities, such as anti‐tumor, anti‐inflammatory, antioxidant, anti‐apoptosis, anti‐bacterial, anti‐atherosclerosis, anti‐angiogenesis, and so on, which determines that it is an appropriate candidate for ideal radioprotectors. Previous studies have shown that RSV reduced the probability of chromosome aberrations in mice bone marrow cells after whole‐body γ‐ray irradiation exposure, and protected mouse embryonic stem cells from IR injury by facilitating the repairment of DNA damages.^[^ ^72^ ^]^ In another recent study, it is shown that RSV could reduce radiation‐induced intestinal injury by inhibiting oxidative stress and apoptosis via the SIRT1/FOXO3a and PI3K/AKT pathways.^[^ ^73^ ^]^ The radioprotective activity of RSV is mediated by multiple interaction mechanisms that need to be completely clarified.

As radiation medicine advances, the mechanisms that cause radiation harm at the biomolecular level are progressively figured out and understood. Certain growth factors are closely associated with radiation‐induced cell death in radiation‐damaged tissues, and they can induce apoptosis after radiation, suggesting that the concept of biological factor inhibition could be leveraged to develop radiation protection drugs. In reality, this design concept has already been taken into consideration and put into practice by researchers, giving birth to new radioprotective drugs. One typical route is to inhibit the p53 transcription factor, according to which some researchers found that sodium orthovanadate (vanadate) exerted a powerful effect on radiation protection, and similarly, zinc Chelation and 8‐HQ derivatives also hold high potentials in radiation protection.^[^ ^74^ ^]^

Despite significant progress has been made in the development of radiation protection medications in both clinical and experimental investigations, the unresolved and facilely overlooked shortcomings remain to be addressed. Amifostine itself has a short half‐life and moderate toxicity, inevitably binging about clinical symptoms including hypotension, nausea, vomiting, weakness, weariness, and drowsiness after taking excessive doses. As a result, its general applicability has always been relatively restricted.^[^ ^75^ ^]^ Similarly, palifermin has side effects such as rash, fever, altered taste, and the risk of cancer, while Neupogen may cause delayed acute respiratory distress syndrome and need to monitor patients' vital signs. All of these problems are recognized as the culprits of application restrictions of radioprotective drugs. In contrast, although many plant extracts have shown excellent radioprotective effects with lower toxicity, most of their applications are also limited due to the poor water solubility, intestinal instability, poor absorption, short circulating time, and low bioavailability.^[^ ^76^ ^]^ For instance, although resveratrol has been widely accepted to implement the antioxidant and anti‐radiation actions, many researchers argue that the studies related to the absorption, metabolism, and bioavailability of resveratrol in humans are still inadequate, and the interactions between its pharmacological effect with other therapies especially such as radiotherapy are still unclear. As a result, the biological mechanisms of its action need to be further determined.^[^ ^77^ ^]^ Additionally, some novel small molecule compounds such as PUMA inhibitors also exhibit high potential for radioprotection, but its exploration in radioprotection is still relatively in its infancy. Regarding this, more studies especially in animal experiments and clinical trials are needed to prove and validate the efficacy, underlying mechanisms, clinical application potential, and probable biological risks of such promising radioprotectors.

## 3 ADVANTAGES AND DESIGNS OF THE INTRINSIC NANO‐RADIOPROTECTANTS

**FIGURE 2:**
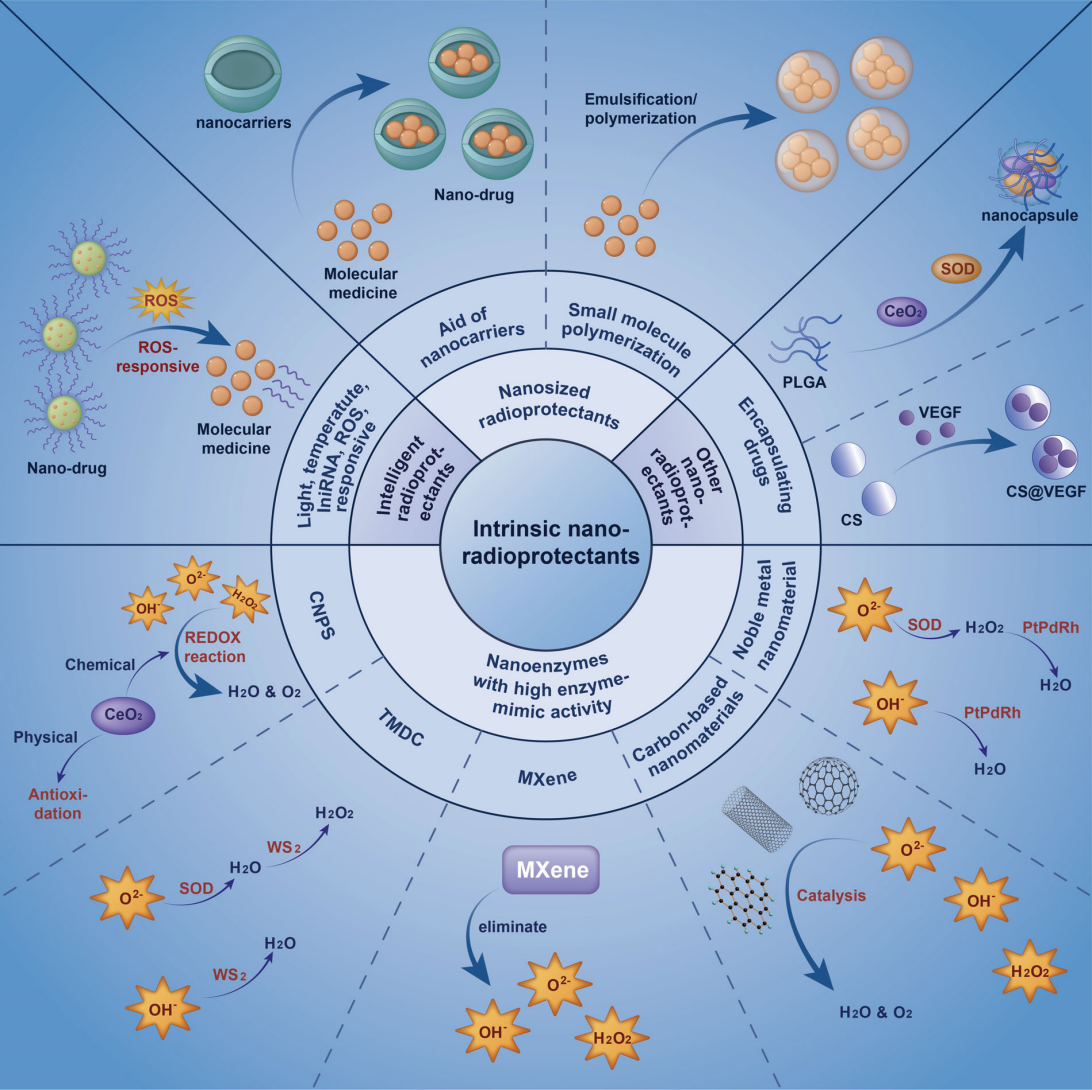
A recent research summary on intrinsic nanoradioprotective drugs. The main categories are illustrated along with their corresponding design principles and representatives. Intrinsic nano‐radioprotectants in this syndrome are mainly divided into four categories including intelligent radioprotectants, nanosized radioprotectants, nanoenzymes with high enzymatic activity, and other nano‐radioprotectants.

To tackle the encountered complex situation using traditional tactics to develop radioprotective drugs, researchers are trying to seek other ways to attain the radioprotective goal, for example, continuously improving and optimizing the traditional drugs. In recent years, with accompanying the continuous breakthroughs and innovations in the field of nanomaterials, more and more attention has been paid to the involvement of nanomaterials in radiation protection, where the increasing discoveries of competent nano‐enzymes offer a promising strategy for radiation protection. Nanomaterials enable radioprotectors to become nano drugs (Figure 2). As radioprotective drugs, nano‐radioprotectors must possess preventive and/or therapeutic functions against radiation damage. By taking advantage of the two aspects above, nanotechnology can help traditional medications overcome a series of inherent problems such as short circulation time and rapid metabolism in the body. Besides, nano drugs are also conferred with other advantages including strong catalytic activity, ease of mass manufacture, long storage time, low cost, and high stability in harsh environments. Benefitting from these merits, carbon nanomaterials such as fullerenes, carbon nanotubes (CNTs), graphene, carbon quantum dots, and graphene quantum dots have become star materials in the radiological biomedical field.^[^ ^78^ ^]^ Another class of well‐studied radioprotective nano‐drugs specifically refers to nanoenzymes, and various biomimetic nanoenzymes are designed and synthesized as effective radiation protectors to simulate the properties of antioxidant enzymes (e.g., CAT, superoxide dismutase (SOD), and Gpx) to eliminate various ROS or RNS and improve the repairment of DNA damages. It is worth noting that the first clinically‐approved carbon nanoparticle suspension injection (CNSI) was recently evidenced that it could also be used as a novel nano‐radiation protection agent for effective intestinal radiation protection. Under oral administration conditions, CNSI displayed powerful radioprotective properties in the intestine with highly‐efficient free radical scavenging ability, good biosafety, strong chemical stability, and relatively long retention time. Consequently, the repair of IR‐induced intestinal flora imbalance was promoted. This opened the door to overcoming radiation enteritis, a serious condition that conventional drugs were unable to cure.^[^ ^79^ ^]^

## 4 RESEARCH STATUS OF INTRINSIC NANO‐RADIOPROTECTANTS

### 4.1 Nanosized drugs

As we all know, nano‐sized objects exhibit remarkable properties in various aspects such as acoustic, optical, electrical, magnetic, thermal, and mechanical properties that are different from those of macroscopic objects, and these properties confer new characteristics to nanomedicines beyond their original chemical properties. Based on these new properties, the nanosized drugs obtained by nanosizing the original conventional drugs are equipped with a series of new advantages, including enhanced bioavailability, improved stability, increased solubility, etc. Many kinds of new nanosized drugs have been developed for antitumor, anti‐inflammatory, and various other applications. Here, we mainly discuss the recent advances in the nanosizing (nanocrystallization) of traditional radioprotectants, which is referred to the polymerization of original radioprotective agents into nanoparticles. As a new research trend, this strategy has been paid great emphasis and made some breakthroughs in improving the efficacy of kinds of medications for treating various radiation injuries, showing its promising potential in the radiation protection area.

#### 4.1.1 Small molecule polymerization of radioprotective drugs

**FIGURE 3:**
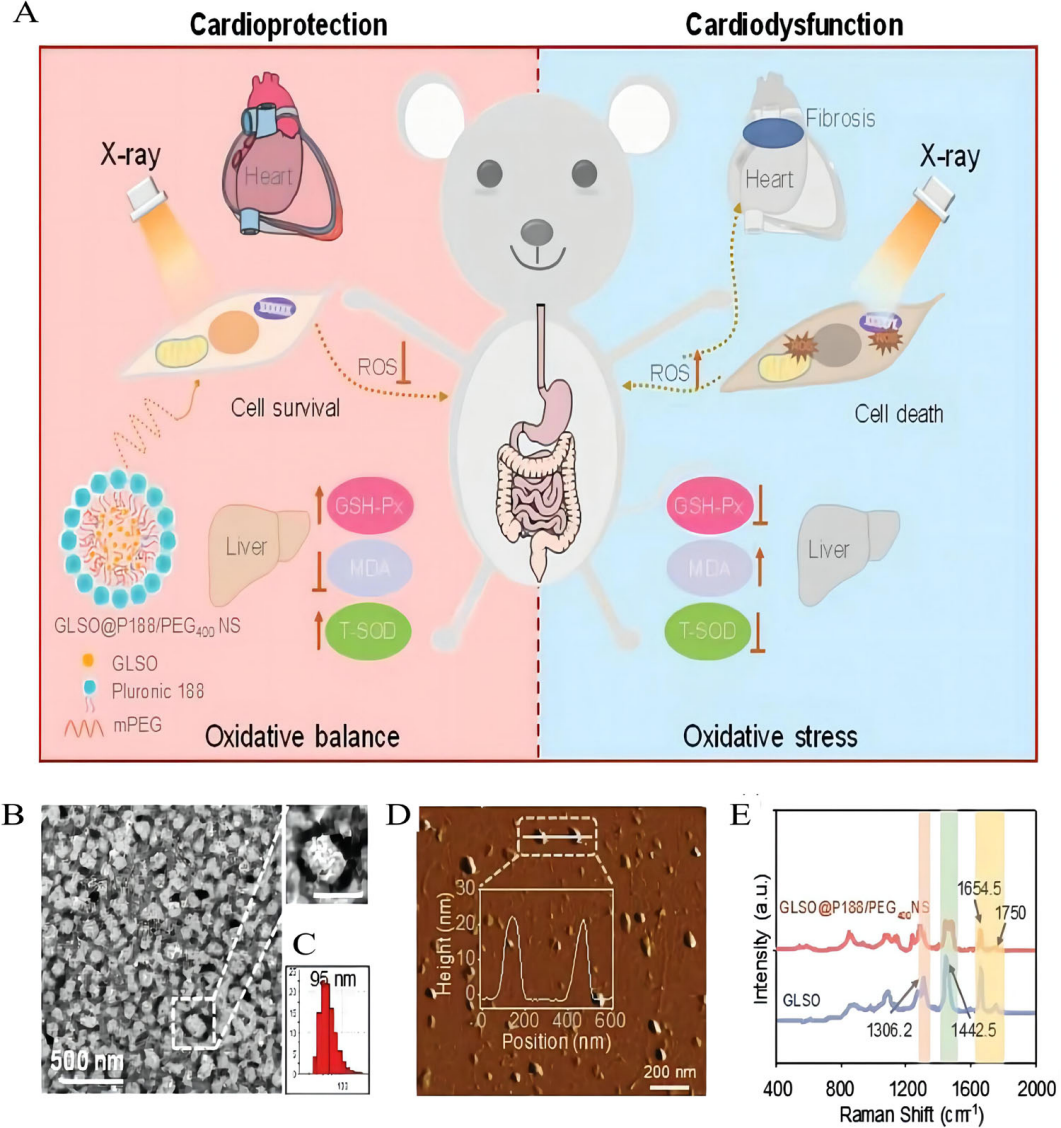
Typical small molecule polymerization of radioprotective drugs. Characterization and illustration for the role of GLSO@P188/PEG400 NS on cardiomyocytes against X‐rays. (A) Schematic illustration for the role of GLSO@P188/PEG400 NS (left section) on cardiomyocytes against X‐rays both in vivo and in vitro. (B) TEM image of GLSO@P188/PEG400 NS, scale bar = 500 and 100 nm. (C) Hydrodynamic size. (D) AFM image (2D) with inserted average height and distance. (E) Raman spectra of GLSO and GLSO@P188/PEG400 NS. Reproduced with permission.^[^ ^81^ ^]^ Copyright 2019, WILEY VCH.

At present, various methods have been used to polymerize radioprotective drugs into nanocrystals, including emulsification, high‐pressure homogenization, and so on. As for emulsification, Silymarin was successfully emulsified into 3–8 nm nano emulsion using surfactants and cosurfactants for liver radioprotection. Compared to the prototypical single silymarin molecules, the radioprotective efficacy was well retained with the bioavailability improved a great deal after the nanocrystallization.^[^ ^80^ ^]^ By using the high‐pressure homogenization method, Chen synthesized nano GLSO@P188/PEG_400_ drug (about 90 nm in size),^[^ ^81^ ^]^ improving the water solubility and further attenuating the generation of excess ROS and better protecting the mitochondria from X‐ray damage (Figure 3). Obviously, this study provides a promising strategy for solving the radioprotectants with good radioprotective abilities but without good water solubility. Also, in some cases, nanocrystallization can be aided by other methods. For example, as conventional radioprotective medication, Lipoic acid (LA) has such a short plasma half‐life that its bioavailability is reduced to roughly 30% of the ingested dose following oral delivery.^[^ ^82^ ^]^ To address this issue, researchers managed to prepare an 11 nm‐sized lipoic acidnanocapses (LANC) and proved its reliable radioprotective potential against ^99m^TC‐MiBi‐induced cardiovascular tissue damage.^[^ ^83^ ^]^

#### 4.1.2 Nanomedicine assembled with the aid of nanocarriers for radioprotection

In addition to assembling radiation protection compounds into nanoparticles, loading the radioprotective molecular onto low‐toxicity nanocarriers is another effective way to improve the intrinsic efficacy and overcome defects such as the low bioavailability. To achieve this goal, the organic nanocarriers chitosan^[^ ^84^ ^]^ and polylactic acid‐glycolic acid copolymer (PLGA) are among the most common avirulent drug carriers of great value.^[^ ^22^, ^85^ ^]^

**FIGURE 4:**
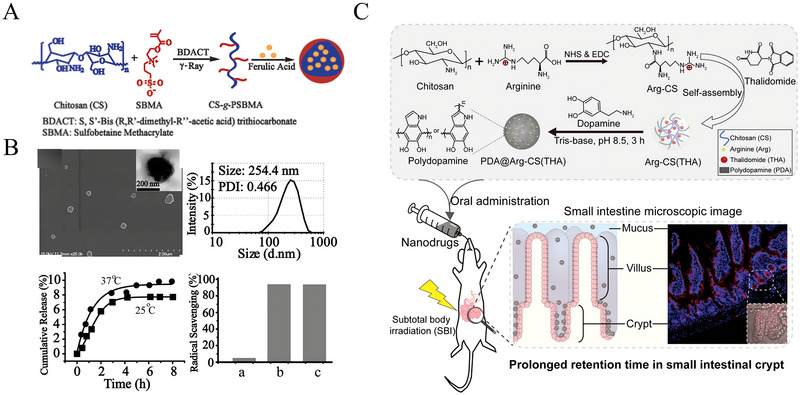
Typical nanomedicine assembled with the aid of nanocarriers for radioprotection. (A) Schematic of the synthesis of CS‐g‐PSBMA (PSBMA; polybetaine sulfonate) nanoparticles for packaging ferulic acid and characterization of CS‐g‐PSBMA. (B) Characteristics of the synthesized CS‐g‐PSBMA nanoparticles. Reproduced with permission.^[^ ^85^ ^]^ Copyright 2015, Royal Society of Chemistry. (C) Schematic of the preparation of PDA@Arg‐CS(THA) and the morphological representative micrograph of the targeted intestine tissue. Reproduced with permission.^[^ ^86^ ^]^ Copyright 2020, Wiley‐VCH.

According to some earlier studies, Ferulic acid (FA) was recognized as an effective anti‐radiation drug, which still has many shortcomings such as low solubility and short half‐life. Recently, Song Hua et al. have synthesized the novel drug delivery system CS(FA)‐g‐PSBMA (255 nm) by wrapping the FA on the CS chain segment that was modified by polybetaine sulfonate (PSBMA) and succeeded in increasing both the solubility and the blood retention time (Figure 4A,B).^[^ ^85^ ^]^ In another study, Daoben Hua et al. provide a new oral radioprotective nanodrug PDA@Arg‐CS (THA) (200 nm) for effectively alleviating the gastrointestinal syndrome caused by IR, which exhibits repressed drug release in simulated gastric acid and pH‐switchable controlled release in the intestinal environment (Figure 4C).^[^ ^86^ ^]^ The benefits of CS nanocrystalline drugs in aiding the radioprotective drugs here are largely due to their capability of increasing the content and prolonging the duration of the drug in the small intestine crypt. Moreover, PLGA is also a commonly used organic polymer used to load radiotherapy protective drugs such as amifostine and penicillamine (D‐3‐mercaptovaline, a metabolite of penicillin).^[^ ^22^ ^]^ Accordingly, the drug efficacy is usually improved via both the elevated drug activity and the strengthened drug solubility.

In addition to organic nanocarriers, inorganic drug carriers are also another important option for carrying radioprotective drugs. For example, most injectable medications are small molecule or cytogenic pharmaceuticals, and they are among the most extensively utilized dose types to alleviate radiation‐induced intestine injury.^[^ ^9^, ^87^, ^88^ ^]^ However, high doses of the injectable medication may instead cause intestine injuries, which can be avoided by delivery based on inorganic drug carriers due to their advantages of stability, high loading capacity, and longer circulation stay in the body. For example, silica, a commonly used nano carrier with low toxicity and biodegradability, was adopted for the delivery of melanin by Schweitzer et al. to synthesize the melanin‐covered nanoparticles.^[^ ^89^ ^]^ As a result, it exhibited a much better protective effect on bone marrow injuries from IR. Besides, Hongqi Tian et al. loaded small molecular radioprotective agents such as WR‐1065 and GSH into metal‐organic framework material Mil‐101(Fe),^[^ ^33^ ^]^ and further modified it through PEGylation. Compared with free drugs, the mucosal penetration, ROS scavenging ability, and circulation half‐life of the MOF‐based drug were significantly improved, while the cytotoxicity was greatly reduced.^[^ ^90^ ^]^

### 4.2 Nanoenzymes with radioprotective properties

As mentioned above, nanoenzymes are designed and synthesized to simulate the properties of antioxidant enzymes which can be harnessed to scavenge various ROS or RNS triggered by IR. ROS are oxygen‐containing and chemically reactive species formed by incomplete one‐electron reduction of oxygen, including hydrogen peroxide (H_2_O_2_), superoxide anion (O^2 −^), hydroxyl radical (•OH), and so on. Such substances often involve heterogeneous electrons that can easily react with other molecules, the phenomenon known as oxidation. ROS are naturally produced in cells through aerobic metabolism, and the mitochondrial respiratory chain, NADPH oxidase, and peroxisome are the main endogenous sources of ROS. Under normal physiological conditions, healthy cells can maintain redox homeostasis by balancing the generation and clearance of ROS, while maintaining low levels of basic ROS, and the moderate increase of ROS was beneficial to cell proliferation and survival.^[^ ^91^, ^92^, ^93^, ^94^, ^95^ ^]^ During radiation therapy, ROS accumulates greatly both in normal tissue and in cancerous cells. In normal tissue, the radiation‐induced ROS quickly breaks the redox balance, leads to the excessive oxidative stress and induces biomolecular damage, and finally cell catastrophe, which manifests the radiation damages to the body.^[^ ^27^ ^]^ As oxidative stress is launched by IR and intermediates its main detriments, reducing the oxidative stress with antioxidant medications has long been deemed an important anti‐radiation strategy. Compared with traditional radioprotectors, antioxidant nanoenzymes featuring higher antioxidant efficacy, stronger stability and greater tolerance to harsh microenvironments have been proven to ameliorate oxidative damages in different radiation disease models.^[^ ^96^ ^]^

#### 4.2.1 Cerium oxide‐based nanoenzymes

In recent years, cerium oxide nanoparticles (CNPs) have shown distinct advantages in the fields of biomedicine and drug delivery, and they have become new protective agents against radiation damage due to their remarkable capacity to scavenge free radicals. Lots of evidence have shown that the protective effect of cerium CNPs mainly originated from their ease of redox reactions in living organisms with the rapid transition of oxidation states between Ce^4+^ and Ce^3+^ (induced by oxygen vacancies). The expansion and contraction of lattice in response to valence change are coupled with the ability to change its valence state and the gain or loss of surface oxygen vacancies. In addition, some researchers indicated that CNPs allowed hydrogen precipitation to neutralize toxic free radicals. Collectively, all of these extraordinary properties guarantee that CNPs can be used as an effective radiation therapy protectant, as confirmed by numerous studies.^[^ ^97^, ^98^ ^]^

Depending on the antioxidant property, CNPs were able to attenuate the human bone marrow mesenchymal stem cells (hBMSCs) damages induced by IR. As hBMSCs were incubated with CeONPs at concentrations of 1 or 10 g·mL^−1^ for 24 h before a single delivery of 7 Gy irradiation, ROS level, cellular proliferation, cell morphology, cell senescence, DNA damage, p53 expression, and autophagy were examined. Furthermore, researchers evaluated the alkaline phosphatase, osteogenic protein gene expression, and bone matrix deposition in differentiated osteogenesis. According to the findings, the cell‐absorbed CeONPs were found to have considerable multifunctional radioprotective effects against cellular damages.^[^ ^99^ ^]^ In addition, CNPs are also characterized by good competence in fault tolerance and readiness for modification. Benefiting from this, their stability can be increased, and their cytotoxicity can be minimized especially after modification with polyethylene glycol or other methods, which further demonstrates the great potential and prospect in the field of radiation protection.^[^ ^100^, ^101^ ^]^ In recent years, researchers have continuously improved the CNPs‐based radioprotectors and expanded their research and application based on their high radiation‐shielding properties. One typical study was hosted by N. R. Popova et al. who demonstrated the radioprotective effect of polyelectrolyte microcapsules modified with CNPs in a controlled loading and intracellular release manner. In this study, the layer‐by‐layer (LbL) method was used to synthesize microcapsules, followed by coating with citrate. During the subsequent radiation protection experiment, the protective ability of microcapsules was confirmed by detecting different pathways of cellular radiology, including apoptosis, necrosis, autophagy, ROS metabolism, and so on. Their results indicated that the CNPs pretreatment managed to rectify the IR‐disturbed gene expression of critical pathways, highlighting the critical role of CNPs in radiation protection.^[^ ^102^ ^]^

#### 4.2.2 Transition metal dichalcogenide‐based nanoenzymes

Completely differing from cerium oxide whose radiation protection relies mainly on the valence change of cerium atoms, transition metal elements have aroused increasing interest because of their inherent active redox reactivity since they emerge first. In contrast with the CNPs, the layered structure of transition metal dichalcogenide (TMDC) that incorporates the transition metal element is unique, and displays as Type MX2, where M represents transition metal elements (Mo, W, Re, Ti, Hf, Nb, Ta) and X represents chalcogenides (S, Se, or Te). In essence, this appealing layer structure is a sandwich‐shaped X‐M‐X layer, with X located between two outer hexagonal planes that were separated by an inner plane M. The atoms in the same layer are covalently bonded, while the adjacent layers are linked by weak van der Waals forces, determining that several layers of TMDC nanosheets can be easily obtained. This structure also favors TMDCs to become flexible and adjustable to form multiple forms.^[^ ^103^, ^104^ ^]^ Moreover, depending on the different combinations and their various arrangements between M and X, more than 40 types of 2D TMDCs were synthesized. This characteristic structure with numerous atomic combinations confers several valuable qualities on 2D TMDCs, including unique electrical and optical capabilities, a vast and easily modifiable surface area, and minimal cytotoxicity.^[^ ^105^ ^]^ Based on these important principles, new radioprotective TDMCs such as WS_2_ and Bi_2_Se_3_ have been developed after innovative and reasonable design, and they show incomparable advantages including high free radical‐scavenging ability, good biocompatibility as well as low biotoxicity.^[^ ^106^, ^107^, ^108^ ^]^

#### 4.2.3 Noble metal‐based nanoenzymes

Since the combination of nanotechnology with radiological biomedical applications emerged, the noble metal nanoparticles have gained researchers' attention, and their first application field is radiosensitization. Herein, gold, silver, and other noble metal elements can absorb X‐ray energy by interacting with IR (such as photoelectric effect, Compton effect, etc.) to emit the secondary electrons, which further act directly on DNA and other biomolecules, or indirectly activate surrounding water to increase the production of free radicals, resulting in radiosensitization of tumor treatment.^[^ ^109^, ^110^ ^]^ Besides, some other noble metal elements such as tantalum, tungsten, and bismuth also have recently shown the effectiveness of radiosensitization. Apart from radiation sensitization, noble metal nanoparticles also play a vital role in the field of radiation protection. Peixian Bian et al. prepared ultra‐small Au‐MoS_2_ clusters by intercalating gold into MoS_2_. Although the sulfide nanomaterials themselves can remove ROS, the synthesized ultra‐small Au‐MoS_2_ clusters gain much better catalytic activity toward H_2_O_2_ than the ultra‐small MoS_2_ without Au. It indicates that this processing effectively enhances the antioxidant activity and radiation protection effect in vivo.^[^ ^111^ ^]^

Pt is another remarkable candidate for developing noble metal nano‐radioprotector. Takeki Hamasaki discovered that Pt nanoparticles were capable of scavenging •O^2−^ and •OH,^[^ ^112^ ^]^ and especially the reasonable surface shape design and appropriate doping were gradually recognized to preferably improve the performance of Pt nanoparticles. Ultra‐small Pt nanoclusters by changing the size of Pt nanoparticles were created and performed as radioprotective agents, and results showed that the irradiated mice treated with Pt nanoclusters received a higher survival rate and increased bone marrow DNA content.^[^ ^113^ ^]^ Subsequently, researchers altered the morphology of Pt nanoparticles and designed and synthesized Pt‐Pd hollow nanocubes using a one‐pot synthesis method, where the zero‐dimensional Pt‐Pd hollow nanocubes had a small plane of high refractive index, thus bringing a larger specific surface and providing more surface‐active centers per unit mass. They discovered that Pt‐Pd nanocubes could serve as the free radical scavenger to boost SOD activity and lower oxidative DNA damages and MDA levels in mice. In addition, Jun‐ying Wang et al. synthesized another class of Pt‐based nano agent, termed as polyvinylpyrrolidone‐protected hollow PtPdRh nanocubes, and these nanocubes successfully elevated cell viability and prolonged the animal survival under large doses of γ radiation exposure. In the subsequent mechanistic investigations, the hollow PtPdRh nanocubes were confirmed to perform as the catalase, peroxidase, and SOD analogs to effectively remove ROS. To further heighten the biomimetic catalysis activity, Wei Long et al. dopped Mo into PtPd nanocrystals to improve its antioxidant activity by dislocating their lattice structure, obtaining PtPdMo. They found that PtPdMo could inhibit intestinal epithelial cell apoptosis, reduce multi‐organ oxidative stress response and improve the number of bone marrow and peripheral blood cells by eliminating ROS accumulation and enhancing DNA repair ability.^[^ ^114^, ^115^, ^116^ ^]^

Elemental manganese (Mn) is also another promising candidate for noble metal nano‐radioprotectors,^[^ ^117^ ^]^ and its catalytic orientation can be adjusted with the change in the surrounding pH condition, thereby increasing its adaptability.^[^ ^25^ ^]^ Stable and efficient Mn_12_ clusters were synthesized by a two‐step reaction between insoluble manganese acetate (Mn (CH_3_COO)_2_•4H_2_O) as an intermediate product and potassium permanganate (KMnO_4_) as oxidizing agents in aqueous acetic acid solution. As expected, the final products exhibited high pH‐dependent catalytic selectivity, and the survival rate of noncancerous cells treated with Mn_12_ clusters was remarkably higher than that of those irradiated alone at the same radiation dose, indicating that Mn_12_ clusters had a strong radiation protection ability in vitro. Also, its high ability to scavenge ROS in the neutral environment of normal cells was observed. In contrast, aiming at cancer cells, Mn_12_ clusters effectively catalyzed ROS generation in the acidic tumor microenvironment, which was validated to further induce apoptosis and necrosis of tumor cells, but improve the total DNA content and nucleated cells of bone marrow under radiotherapy. Therefore, this study provided an unprecedented dual‐functional strategy to enhance tumor radiotherapy efficacy by simultaneously improving tumor oxygenation when protecting normal tissue from radiation.^[^ ^118^ ^]^

Researchers also tried to combine noble metals with other nanomaterials to realize enhanced radioprotection. Sang Ihn Han et al. deposited manganese ions on the surface of cerium oxide (CeO_2_) to create the strain layers of manganese oxide (Mn_3_O_4_) island and increased the number of oxygen vacancies to improve their radioprotective performance. It was indicated that the composite CeO_2_/Mn_3_O_4_ nanocrystals showed greater catalytic activity than CeO_2_ alone or Mn_3_O_4_ alone, reaching more potent radioprotective effects on intestinal stem cell regeneration even though they received a fatal dose of radiation. Moreover, low‐dose nanocrystals could prevent severe ARS in mice with a considerably‐improved survival even when those mice were exposed to a deadly dosage of total body radiation.^[^ ^119^ ^]^

#### 4.2.4 Carbon‐based nanoenzymes

In recent years, carbon‐based nanomaterials have ignited a surge in many fields, and the unsaturated bonds in their structures unexpectedly make them attractive in the biomedical field. Since the unsaturated bonds can react strongly with free radicals and scavenge them, the design and development of new radiation protection agents based on carbon‐based nanomaterials are becoming more and more prevalent. Currently, several carbon‐based nano‐radioprotectants have been reported including fullerenes in zero dimension, CNTs in one dimension, graphene in two dimensions, and other representatives. In this section, the recent accomplishments in terms of carbon‐based nano‐radioprotectants were summarized and their design concepts were elucidated.

Fullerenes as the typical zero‐dimensional allotropes of graphite carbon were discovered in 1985, and they are closed, featuring of hollow cage consisting of carbon atoms with all SP^2^ hybrids arranged in 12 pentagons and a computable hexagon number. When the number of pentagons reaches 12, a simple spherical molecule with an outer diameter of 0.71 nm can be obtained and called C60, which is chemically similar to an organic molecule.^[^ ^120^ ^]^ In addition to C60, fullerene derivatives such as C70, C80, and C94 can be prepared as the number of carbon atoms varies. Researchers found that although C60 is highly nonpolar, its solubility in common organic solvents and water is both very low, which severely limits its potential biological applications. However, as the relevant study moved forward, researchers managed to attach various water‐soluble groups onto the surface of fullerene molecules, or instead, directly wrap fullerene C60 in the hydrophobic space of hydrophilic substances such as cyclodextrin and calixarenes enhancing the biological application of C60.^[^ ^121^, ^122^, ^123^ ^]^

To overcome the disadvantage of complete insolubility of fullerene C60, a variety of polar functional groups or molecules have been attached to them. These approaches succeeded in retaining the inherent properties of fullerene and achieved reasonable bioavailability. For example, carboxyl groups (─CHCOOH)‐functionalized fullerenes C60(CHCOOH)_2‐6_ introduced from, amino group (─NH_2_)‐chelated fullerene derivatives and hydroxylation fullerenol (C60(OH)n) have been widely studied,^[^ ^115^, ^124^, ^125^, ^126^ ^]^ among which fullerenol C60(OH)n can be engineered into water‐soluble one when the number of hydroxyl groups exceeds some threshold value. Water‐soluble fullerols containing conjugated double bonds possess high polarity and electron affinity, strong ROS capturing ability, and ideal free radical attachment activity, which thus can meet the basic requirements of radioprotective chemicals and draw much attention in the field of radiation protection. Among them, C60(OH)_24_ has been deeply and comprehensively studied after long‐term research and explorations, and researchers have identified a suitable concentration range for C60(OH)_24_ to exert the most effective radioprotection.^[^ ^127^, ^128^, ^129^ ^]^ Jacek Grebowski et al. conducted another study and fabricated C60(OH)_36_ with strong radioprotective ability against human erythrocyte membranes. Through systemically investigating indicators capable of reflecting the blood cell membrane function, for example, potassium ion release level from erythrocytes, membrane mobility change, and membrane protein conformational alterations, they confirmed the ability of C60(OH)_36_ to reduce the damage to the plasma membrane of human erythrocytes induced by 6 MeV energetic electrons.^[^ ^130^ ^]^

Beyond this, many researchers managed to develop fullerene molecules with different forms other than chemical group modification. Sergey V. Gudkov et al. studied unmodified hydrated C60 fullerene molecules (C60UHFM) and found that C60UHFM could reduce ROS birth in irradiated water, and enable treated mice to be liberated from IR‐induced crisis in animal experiments by reducing the DNA damage, relieving the loss of leukocytes and platelets, and boosting the regeneration of small intestine tissues.^[^ ^131^ ^]^ Recently, another team synthesized water‐soluble Fullerenol @ nano‐montmorillonite (NMMT) by mechanochemical synthesis, and the obtained NMMT displays high chemical stability and extensive radical scavenging ability, which could effectively mitigate the damages to mitochondria and DNA caused by IR‐generated ROS. Intriguingly, NMMT as a drug carrier was equipped with a strong intestinal adhesion capacity, and allowed a longer residence period in the duodenum for fullerenol, thus reducing radiation‐induced diarrhea, weight loss, and histopathological damage in the duodenum of mice.^[^ ^132^ ^]^

From the perspective of spatial dimension, fullerenes are classified into zero‐dimensional materials, while the well‐known CNTs are one‐dimensional ones. Compared to zero‐dimensional nanoparticles such as fullerenes, one‐dimensional nanoparticles have not been fully studied in the field of radiation protection yet. The linear structure of one‐dimensional nanomaterials is the most typical characteristic that was clearly distinguished from zero‐dimensional nanoparticles. CNTs are believed to be one of the most prominent materials, and they have been frequently employed in biochemistry due to their advantages including fibrous shape, high porosity, ease of production, and outstanding mechanical strength. It has been reported that CNTs can either generate or scavenge ROS in numerous studies, endowing them with the ability to protect cells against radiation damage.^[^ ^133^, ^134^, ^135^ ^]^ In addition, CNTs have other distinctive properties such as excellent cell permeability, making CNTs appropriate for building drug delivery systems, and a series of successful cases have validated it. Moreover, many attempts have been made to functionalize CNTs with functional groups such as oxygen, nitrogen, and hydrogen atoms, thus reducing their toxicity and increasing their solubility and drug delivery. This strategy has been widely accepted in the field of drug delivery and potentially provides new solutions to improve the performance of radiation protection drugs.^[^ ^136^, ^137^ ^]^

The development and application of two‐dimensional nanomaterials for radiation protection have brought more impressive achievements than one‐dimensional nanomaterials, among which, carbon‐based two‐dimensional nanomaterials such as graphene are the most representative. Graphene oxide (GO) is a water‐soluble, non‐toxic, biodegradable, and modifiable nanomaterial, where all carbon atoms of GO are exposed and the carbon atoms at the edges are more reactive than those in the plane. The open structure of GO allows it to efficiently trap oxygen radicals with carbon atoms at the edges. Given this, the ability of GO to act as a free radical scavenger for radiation protection is expected.^[^ ^138^ ^]^ Meanwhile, the ease of modification of graphene is also advantageous for optimizing radioprotectors. As a paradigm, Junying Wang et al. designed a kind of monolayer graphene‐coated metal nanoparticles (MGMN) to remove free radicals by electron transfer between monolayer graphene and metal nuclei. After systematical assessments including SOD, DNA damages, ROS, and animal survival, the as‐prepared MGMN potentially served as a potent IR protector.^[^ ^115^ ^]^

The aforementioned fullerenes and graphenes consisting of benzene‐type carbon networks have been documented to possess good biocompatibility and strong scavenging activity of free radicals. A scrutiny of their functional‐structural commonalities reveals that these biological activities derive from the conjugation system in their molecular structures. According to this principle, researchers started to pay attention to graphyne, another new class of carbon network materials composed of benzene and alkyne groups. Similar to graphene, graphyne could be used to engineer new radioprotective drugs because of its strong conjugated π‐system and highly reactive diacetyl bonds, which provide a powerful scavenging ability of free radicals. For example, Jiani Xie et al. fabricated the bovine serum albumin (BSA)‐modified graphdiyne (GDY) nanoparticles (GDY‐BSA NP) and investigated its gastrointestinal radiation protection capability. In vitro and in vivo experimental results showed that GDY‐BSA nanoparticles could effectively mitigate DNA damages in gastrointestinal cells and improve cell survival after irradiation, and significantly alleviate radiation‐induced diarrhea, weight loss, and gastrointestinal pathological damages in mice. Moreover, the mechanistic studies showed that GDY‐BSA accomplished radioprotection by effectively inhibiting ROS‐induced apoptosis in gastrointestinal cells.^[^ ^139^, ^140^ ^]^ Besides, the nanosized graphdiyne‐loaded sodium hyaluronate hydrogel (nano‐GDY@SH hydrogel) was equipped with high biosafety and strong broad‐spectrum scavenging activity of free radical, and the as‐prepared nano‐GDY@SH hydrogel was also proved to effectively relieve low‐energy X‐ray‐induced skin injuries, remit skin edema and ulcer, accelerate skin damage repair and promote wound healing.^[^ ^141^ ^]^ Exogeneous and endogenous triggers such as temperature arm nanoparticles or hydrogels with stimuli‐responsive function, could be engineered into smart drug delivery systems and enable the on‐demand release of drugs or functional molecules for imaging and treatment of lesions.^[^ ^142^, ^143^, ^144^ ^]^

#### 4.2.5 MXene‐based nanoenzymes

Encouraged by the in‐depth and comprehensive understanding of two‐dimensional (2D) nanomaterials based on the representative graphene, many 2D nanosheets were explored to realize radioprotective actions because of their large special surface area‐to‐mass ratio and high cargo‐loading structure. Titanium carbide as a new carbon‐based material that was obtained by etching aluminum titanium carbide using the mixture consisting of fluoride salts and either concentrated hydrofluoric acid (HF) or hydrochloric acid (HCl) has been well studied and has shown great potential in the biomedical field.^[^ ^145^ ^]^ Additionally, other carbide‐based nanosheets such as vanadium carbide, and niobium carbide also have been developed using the identical principle and method, where niobium carbide nanomaterials exhibited efficient photothermal ablation and tumor eradication in vivo in some studies.^[^ ^146^ ^]^ Very recently, an ultrathin 2D niobium carbide (Nb_2_C) was developed as a new class of radio‐protectants and showed the high scavenging ability of ROS involving hydrogen peroxide (H_2_O_2_), hydroxyl radicals (•OH), and superoxide radicals (•O_2_^−^).^[^ ^147^ ^]^ Especially after the polyvinyl pyrrolidone (PVP) modification, the obtained Nb_2_C‐PVP outperformed Nb_2_C in scavenging ROS, improving survival rate, attenuating the radiation damage to the hematopoietic system, and improving the pathological damages in the testis, small intestine, lung, and liver of irradiated mice.^[^ ^148^ ^]^

Generally, nanomaterials with two‐dimensional structure, for example, titanium carbide and niobium carbide, are also termed as MXene with a common chemical formula: M*_n_*_+1_X*_n_* T*_x_* (n = 1∼3), where M and X represent the early transition metal and C or N elements, respectively, and T*_x_* refers to the surface group. Recently, MXene material exhibited a wide application domain in the field of biomedicine. As a paradigm, tantalum carbide and titanium nitride nanoparticles as ideal light absorbers could be engineered to achieve multi‐imaging‐guided photothermal tumor ablation, determining that they were also appropriate for photothermal therapy and photoacoustic imaging due to their outstanding biocompatibility and bio‐transparency.^[^ ^149^, ^150^ ^]^ Furthermore, Feng et al. designed and synthesized a vanadium carbide enzyme that mimicked up to six natural enzymes. This summary unveils that MXene not only has high bio‐compatibility but also holds a strong protective effect on cells and tissues from oxidative injuries including IR stress.^[^ ^151^ ^]^

### 4.3 Stimuli‐responsive radioprotective nanodrugs

The intelligent nanomaterials, also called stimuli‐responsive nanoparticles, are referred as the nanosystems with the ability to respond to environmental alterations such as light,^[^ ^152^ ^]^ pH,^[^ ^153^ ^]^ temperature,^[^ ^154^ ^]^ miRNA,^[^ ^155^ ^]^ enzymes,^[^ ^156^, ^157^, ^158^ ^]^ ROS,^[^ ^159^ ^]^ and so on. As can be seen, these technologies have been applied in many areas and exert very important promotive effects in health science. Nevertheless, in the radioprotective field, the application of this technique is relatively rare, with most research devoted to the ROS response to radiation, which will be discussed mainly below.

**FIGURE 5:**
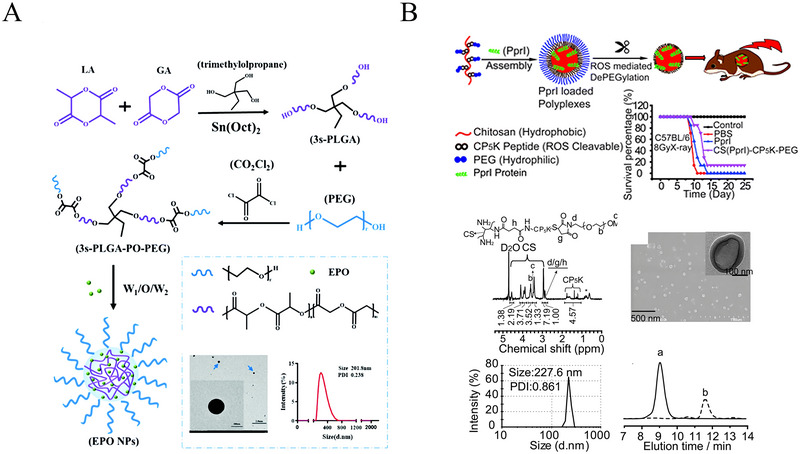
Typical ROS‐responsive radioprotective drugs. (A) Preparation of EPO NPs. Reproduced with permission.^[^ ^162^ ^]^ Copyright 2021, Royal Society of Chemistry. (B) Schematic illustration of the synthesis route of CS (PprI)‐CP5K‐PEG and characterization of CS (PprI)‐CP5K‐PEG. Reproduced with permission.^[^ ^164^ ^]^ Copyright 2018, Royal Society of Chemistry.

ROS is regarded as a pivotal signaling molecule involved in many physiological processes and contains many key mediators in the cellular functional biological activities.^[^ ^74^, ^160^ ^]^ Excessive ROS production may disrupt cellular homeostasis, causing nonspecific tissue damage and leading to a series of diseases.^[^ ^15^, ^161^ ^]^ Thus, the ROS‐responsive nanocarriers, which respond to the excessive ROS in cells, have a wide range of potential applications including ROS detection, scavenging, drug delivery, protection from oxidative damage, and so on. As mentioned above, the IR‐injured tissues generate lots of excessive ROS, which are deemed as the upstream trigger events of IR response.^[^ ^15^, ^34^ ^]^ In recent years, the design of ROS‐responsive biomaterials for radioprotection has been identified as a promising therapeutic approach. ROS‐responsive nanodrug can be degraded to release pharmaceuticals in reaction to ROS, which can be employed as a site‐specific delivery method for radioprotection. For instance, Yang et al. prepared a ROS‐responsive polymer, 3s‐PLGA‐PO‐PEG(PP), which can encapsulate EPO with excellent loading capacity and stability and remove excess intracellular ROS efficiently in response to the specific high ROS sites (Figure 5A).^[^ ^162^ ^]^ In addition, researchers used the ROS‐response linker thioketal, which can induce a cascade activation reaction under the ROS stimulation, to deliver WR‐1065 and fabricated the drug‐loaded nanoparticles (PCEC/WR‐1065 NPs). The result shows that PCEC/WR‐1065 NPs can prevent the drug from being destroyed by gastric liquid and intelligently release WR‐1065 in the ROS lesions, resulting in survival benefits, hematopoiesis alleviation, and radio protection of main organs.^[^ ^163^ ^]^ As another example, the CS (PprI)‐CP_5_K‐PEG complex designed by Zhang et al. also exhibited excellent radiation protection performance (Figure 5B). In this research, the PprI protein, which holds the great ability to mitigation for IR injuries, was loaded into the CS‐CP_5_K‐PEG backbone. The newly synthesized nano‐PprI drugs not only helped the free PprI protein overcome the shortcomings such as low stability, short half‐life, and high immunogenicity but enable it to be easily released and ready to enter the damaged tissues according to the tendency of its polypeptide to break up under high ROS circumstances. As confirmed by their data, the nano‐PprI drugs increased cell viability, overall animal survival, and remarkable radioprotection for the hematological system.^[^ ^164^ ^]^ Though many studies of this field are emerging on and on, there is still a long way to go for developing high effective ROS responsive radioprotectants, which should be further strengthened in the future.

Besides, the pH‐responsive and other kinds of radioprotective nanodrugs have also been paid much attention to. As mentioned above, the Mn‐based radio modifiers are typically pH responsive nanodrugs which can diverge its catalytic orientation relevant to ROS level at different acidity, making them ideal dual‐functional radio modifiers in radiotherapy practice. In general, more and more of the stimuli‐responsive radioprotective nanodrugs are being investigated in recent years, greatly increasing the appeal of this young area.

### 4.4 Other intrinsic nanodrugs for radiation protection

The above cases focused on utilizing the inherently antioxidant abilities of nanomaterials to achieve the goals of free radical scavenging and radiation protection. Other properties of nanomaterials such as large pore size and long blood retention time also can be leveraged to deliver the traditional radioprotective drugs. In this regard, lots of candidates as drug vehicles including fullerenes, CNTs, GO, chitosan, MOF materials, etc., show good delivery performance. Along with this approach, the combined nano drugs in nanocarriers always evoked more potent radioprotection. Lin et al. encapsulated radioprotective drugs such as WR‐1065 within PEG‐PCL nanoparticles to reduce the cytotoxicity of amifostine, improve its digestive tract stability, and control its release to enhance the radioprotective activity.^[^ ^151^ ^]^ Jian Cao et al. also adopted various MOF materials to encapsulate WR‐1065,^[^ ^165^ ^]^ and Hussein E. Ali loaded resveratrol with CNTs and resulted in a better‐controlled release, thus greatly improving the therapeutic potential of original drugs.^[^ ^166^ ^]^

Besides encapsulating drugs, some other inspiring strategies have been presented. For example, Apoorva Mehta used PLGA to encapsulate nano‐cerium oxide and SOD to reduce the original drug's toxicity. Also, Daojiang Yu et al. developed VEGF chitosan nanoparticles by combining chitosan nanoparticles with vascular endothelial growth factor (VEGF) to improve local microcirculation and enhance the protective effect on vascular endothelial cells and skin injury.^[^ ^167^, ^168^ ^]^ As more and more successful attempts to use intrinsic nanomaterials for radiation protection arise, we believe that there is still much space left for acquiring more innovations in this interesting and promising area.

## 5 THE EXISTING KEY COMMON SHORTCOMINGS OF INTRINSIC NANO‐RADIOPROTECTIVE AGENTS

### 5.1 Toxicity

As we all know, toxicity and biosafety of newly‐developed drugs are always one of the most important concerns that researchers need to consider. Since the advent of nanomaterials, the toxicity effects and potential damages of nanomaterials to the body were discussed. Conclusively, it was agreed that nanomaterials were somewhat toxic to the body under certain conditions, and the toxicity was influenced by many aspects, which were generally divided into two major levels: one is the inherently physical and chemical properties of nanomaterials, and another is the interactions between nanomaterials and organism. As for the physical and chemical properties, the particle size, shape, and surface charge of nanomaterials are the main factors capable of affecting toxicity, which should be carefully taken into consideration by designers at the beginning of their research. It is well known that smaller size and a larger specific surface area means a better job of nanomaterials in the biomedical field, but these properties can also increase toxicity and disfavor biosafety.^[^ ^169^ ^]^ Typically, the biological effects of gold nanoparticles with different particle sizes on liver tissues were investigated to find out the toxicity pattern in the treatment, and it was found that the smaller the particle size approached, the greater the toxicity caused.^[^ ^170^ ^]^ After summarizing the relevant literature in recent years, although the size variation of gold nanoparticles ranging from 2 to 10 nm was not found to correlate with the magnitude of toxicity, it is still evident that larger gold nanoparticles (5 to 100 nm) were less toxic.^[^ ^171^ ^]^ Actually, in addition to noble metal nanoparticles, non‐noble metal nanoparticles also agreed with the same pattern. Raziye Mohammadpour et al. observed that the 50 nm diameter silica nanoparticles were more toxic than the 500 nm diameter silica nanoparticles (SNPs) when they were injected intravenously into mice.^[^ ^172^ ^]^

The impacts of morphology on nanomaterial toxicity are complicated and diverse. Compared with other types of nanomaterials that are mostly shaped into particles or clusters, carbon‐based nanomaterials tend to shape into various morphologies. Regarding this, the toxic effects of carbon‐based nanomaterials are diversified because of their various morphology variations. CNTs have a high aspect ratio for benefiting drug encapsulation, but they also pose a great potential threat to living organisms.^[^ ^44^, ^173^, ^174^ ^]^ In another case, 2D layered graphene also exhibits different toxic levels depending on the number of layers, lateral dimensions, hardness, hydrophobicity, and other parameters.^[^ ^175^ ^]^ Apart from the carbon‐based nanomaterials, Rong Pan et al. conducted bactericidal experiments by designing and synthesizing three forms of nano tritonates and found that the changes in shape and length remarkably affected the biotoxicity of nano tritonates.^[^ ^176^ ^]^ Besides particle size and morphology, the surface charge of nanoparticles is also a determining factor of biosafety since it can affect various physiological processes such as cellular adsorption, substance uptake, and cellular internalization.^[^ ^177^ ^]^ In a study focusing on inorganic nanomaterials, researchers found that different surface charges conferred quantum dots with different cytotoxicity, for example, quantum dots with positive charges showed higher cytotoxicity than those with negative charges.^[^ ^178^ ^]^ It is noteworthy that the pattern of charge effects on nanomaterials is not invariable, which is associated with the type of nanomaterial. Researchers have discovered that gold nanoparticles are more likely to disrupt cell membranes when they are charged with cationic electricity rather than negative or neutral electricity. On the contrary, the negatively charged surface functionalization of CNTs showed stronger toxicity than positively charged ones after acid washing.^[^ ^179^, ^180^ ^]^

In addition to the structure‐based influencing factors mentioned above, the interactions between nano drugs and organism metabolism, that is, the biological mechanisms, also take the responsibility for biotoxicity origin. Cell membrane and critical organelles that are responsible for controlling the passage of substances into and out of cells are the first barriers that nanomaterials encounter when they enter the target tissue. The cell membrane is very vulnerable to toxic stimuli including nanomaterials, and a typical representative is the nanosilver that can interact with cell membranes to kill bacteria.^[^ ^181^ ^]^ Cheng et al. incubated fibroblasts with silver nanorods with varying concentrations for 24 h and found that many damage manifestations (e.g., roughening, multiple hollow structures, wrinkling, and even apoptosis) occurred to the treated cell membranes in sequence as the concentration of silver nanorod treatment increased.^[^ ^182^ ^]^ In some other studies, it has been found that the irregular shape and rough surface of CeO_2_ nanoparticles could directly adhere to the surface of cell membranes and disrupt the integrity of cell membranes, thus disturbing the process of substance exchange between cells and extracellular matrix environment and altering the adhesion and fluidity of cell membranes.^[^ ^183^, ^184^ ^]^ These damages not only directly caused cell death but also brought about the function reduction or loss of cell membrane in managing the entry and exit of substances.

Although some nanomaterials can cause structural and functional damage to cell membranes, their inherent small size makes them easily engulfed by cells through certain pathways including phagocytosis, cytokinesis, lattice‐protein‐mediated or niche‐mediated endocytosis, and perforation in biological membranes.^[^ ^185^, ^186^ ^]^ It is found that nanomaterials can cause cell damage by raising ROS levels during or after cell ingestion. For instance, Zhang et al. measured intracellular ROS production in the immunotoxicity test of bone‐marrow‐derived mesenchymal stem cells (BMSCs) after incubation with high‐dose GO, and they discovered that ROS levels were significantly increased at 2.5 and 12.5 μg·mL^−1^ groups.^[^ ^86^ ^]^ Similarly, Peihuan Wang et al. detected the production of ROS in mouse osteoblasts by flow cytometry and found that ROS levels in mouse osteoblast cell line Mc3t3‐e1 treated with tantalum nanoparticles were significantly increased compared with those in the control group.^[^ ^187^ ^]^

Under physiological conditions, ROS are continuously manufactured by the electron transport chain and simultaneously scavenged by the antioxidant system consisting of SOD, CAT, and glutathione. When nanoparticles enter cells, they not only facilitate ROS production but also interfere with the activity of antioxidant enzymes, thus disrupting the redox balance and causing cell damage. Sania Naz et al. pointed out that copper oxide nanoparticles could increase ROS levels and interfere with the normal levels of antioxidants such as catalase, SOD, glutathione peroxidase, and glutathione in cells once they entered cells, which thereby resulted in oxidations of lipids, proteins, and DNA and posed cellular damages.^[^ ^188^ ^]^ Another study on mouse hippocampal neurons showed that titanium dioxide nanoparticles with a certain concentration caused a considerably‐increased ROS level and triggered robust oxidative stress responses, leading to mitochondrial damages represented by enhanced mitochondrial membrane permeability, decreased mitochondrial membrane potential (MMP), and elevated adenosine triphosphate (ATP) levels.^[^ ^146^ ^]^ Notably, a large number of documents recorded that the redox imbalance triggered by intracellular nanomaterials could further induce damage to the structure and function of a variety of organelles such as mitochondria, lysosomes, and endoplasmic reticulum through different pathways.^[^ ^189^, ^190^, ^191^ ^]^ Ping Dong et al. observed the biological effects of BSA‐wrapped ultra‐small silver nanoparticles (Ag‐BSA‐NCs) on mitochondria and found that Ag‐BSA‐NCs induced membrane permeability conversion by interacting with the phospholipid bilayers of mitochondrial membrane and generated ROS to disrupt the mitochondrial respiration associated with mitochondrial damages.^[^ ^192^ ^]^ Additionally, in another study wherein the primary cultured mouse peritoneal macrophages were incubated with the CNTs and GO, the two carbon nanomaterials were accumulated in the lysosomes of macrophages, leading to lysosomal membrane destabilization and posing adverse effects on the cells.^[^ ^193^ ^]^ In addition to the above‐mentioned direct mechanism of ROS production, researchers found that some other nanomaterials are absorbed by phagocytes such as macrophages and neutrophils in the body, and then stimulate them to explosively produce large amounts of ROS to destroy the nanomaterials and simultaneously cause cell damages. As a classic example, Polyhexylcyanoacrylate nanoparticles caused macrophages to erupt in large quantities of ROS, and this process was well known as secondary oxidative stress.^[^ ^194^ ^]^

According to the previous discussion, it is clear that nanomaterials can trigger oxidative stress in cells through direct and indirect pathways, and the activated ROS accumulation can cause damage to various organelles such as mitochondria and lysosomes, which will eventually induce critical cellular events such as cellular autophagy. Autophagy is an important multi‐molecularly regulated biological process, during which pathogens, cellular proteins, or even whole organelles are transported into lysosomes for degradation. The degraded components are in turn recycled to generate new cellular structures or organelles, or else they can be further processed and used as a source of energy. Autophagy can be initiated by a variety of stressors such as energy stress, IR stress, etc. Although it can recycle senescent, damaged cellular components to nourish cells, it can also lead to cellular destruction in some circumstances.^[^ ^195^ ^]^ In an early study, Hideyuki Yamawaki et al. found that C_60_(OH)_24_ promoted autophagic cell deaths when exploring the effect of C_60_(OH)_24_ on vascular endothelial cytotoxicity, and confirmed the increased LC3 II level through Western blot analysis.^[^ ^196^ ^]^ In recent years, with the significant development of molecular biology techniques, Ersin Demir et al. found that C60 could be used to regulate the levels of caspase‐3, Bcl‐2, beclin‐1, and LC3I/II, which in turn improved the intracellular redox imbalance and hyperglycemia‐induced disorders of apoptosis and autophagic flux, exerting a protective effect on cells.^[^ ^21^ ^]^ Therefore, it should be emphasized here that nanomaterials indeed can induce autophagy, and the autophagy‐induced outcome may be either beneficial or harmful to cells, depending on different conditions. The damages caused by nanomaterials‐induced autophagy are discussed here. There are many signaling pathways to regulate autophagy, among which the classic one is the mammalian target of rapamycin (mTOR) signaling pathway. mTOR is a highly conserved serine/threonine‐protein kinase, which is vital for nucleating two distinct protein complexes, called mTOR complex 1 (mTORC1) and mTOR complex 2 (mTORC2). mTORC1 can control protein synthesis, while mTORC2 is capable of regulating cytoskeleton and cell survival signaling. Concurrently, many different signaling pathways (e.g., PI3K/Akt) can regulate mTOR, where Akt as an upstream activator of mTORC1 is a serine/threonine protein kinase and PI3K is an Akt stimulating factor. In detail, the abnormalities of PI3K or Akt will affect the initiation of autophagy.^[^ ^197^ ^]^

Accumulative studies have been conducted to uncover the relationship between nanomaterials and autophagy‐associated cytotoxicity. Lihua Ren et al. found that silica nanoparticles (SiNPs) could reduce the expression of AKT, activate the AMPK/TSC/mTOR pathway, induces autophagy, and cause spermatocyte toxicity.^[^ ^198^ ^]^ Xiaofei Zhou et al. found that the WS2 nanosheets activated the dephosphorylation of mTOR and thus induced autophagy via inhibiting the expression of autophagy inhibitors CXCR‐4 and IGF‐1 no matter where they reside inside or outside of cells, increasing the feasibility of safe use of 2D nanomaterials.^[^ ^199^ ^]^ AgNPs were also verified to trigger cytotoxicity of HT22 cells (mouse hippocampal neuronal cell line) via up‐regulating autophagy‐associated phosphorylation ratios of PI3K, AKT, and mTOR for activating autophagy and apoptosis of HT22 cells.^[^ ^200^ ^]^ In the widely studied and applied carbon‐based nanomaterials, the same problem still exists. It has been confirmed that graphene quantum dots (GQDS) carrying amino groups have a significant inhibitory effect on Akt,^[^ ^201^ ^]^ and GO could regulate the PI3K/Akt/mTOR pathway to enhance the autophagy, and identical phenomena were also observed in other carbon‐based nanomaterials.^[^ ^177^ ^]^ In truth, different cellular signaling pathways are always interplayed to each other in a complicated manner, and the autophagy pathway is not an exception. In terms of nanomaterial‐induced autophagy, it is frequently tightened to the apoptosis process. To figure out the relationship, the cytotoxicity of CNPs with different sizes was investigated on human lung epithelial cells (BEAS‐2B). It was obtained that as cerium oxide entered cells, the intracellular ROS was increased to trigger the activation of caspase‐3 and chromatin condensation, resulting in cell apoptosis.^[^ ^202^ ^]^ In another study, investigators surveyed how manganese nanoparticles activated mitochondria‐dependent apoptosis and autophagy in neuronal cells. A significant increase in intracellular ROS level was induced when manganese nanoparticles entered the cells, which further triggered oxidative stress responses, prompted the up‐regulations of beclin‐1 and LC3 proteins, and activated the caspase‐3‐mediated apoptosis.^[^ ^203^ ^]^ Identical results were found in bismuth (Bi)‐based nanoparticles such as bismuth nanoparticles, bismuth nitrate, chlorooxy bismuth, bismuth citrate, and bismuth subcitrate.^[^ ^204^ ^]^

### 5.2 Relatively limited research depth

Up to now, although mounting cellular and animal experiments have shown the great potential of nanomaterials in radiation protection, there is still a huge distance between the real large‐scale application of nanomedicines in radiation protection, and the biomedical research of radioprotective nanomaterials is still at its early stage. For example, from Table 1 we can see that though multiple studies targeting different organs have been extensively carried out, the exact mechanism remains largely to be elucidated. As for side effects, a large number of experimental studies have proved that nanomaterials could induce oxidative damage and inflammation, and activate different signaling pathways such as cellular autophagy. However, the exact mechanism is still not elucidated, which is one of the main obstacles that limit the future applications of nanomaterials. Encouragingly, some researchers have undertaken more detailed studies on the toxicity of certain nanomaterials. For example, researchers recognized that Ce^3+^ content in CNPs not only generated toxicity by increasing ROS production but also interfered with their adherence to cells, thereby influencing the toxicity, which was determined by many factors such as aspect ratio, surface charge, entry rate, cell culture environment, cell type, and storage conditions.^[^ ^169^ ^]^ In addition, no matter what noble metal nanomaterials were developed and applied, the most advanced experiment exploration is only limited to the animal model, and few were verified at a high level such as organoid or clinical experiments. This discourages researchers to expand the application of nanomaterials in the biomedical field. Therefore, there is still a long way to go to promote the in‐depth research and application development of nano‐radioprotectants.

**TABLE 1:** A summary of reported nanomaterials for radioprotection targeted to various organs

Organ	Mechanism of radiation injury	Nano‐radioprotective drugs	Protective principle	References
Heart	PPARα inactivation, fatty acid oxidation (FAO) decreased Inflammatory response Mitochondrial ROS levels increased Signaling pathway inactivation	GLSO@P188/PEG400; 99mTc‐MIBI	The antioxidant effect of the molecule drug	[81, 83]
Liver	Complex and multicellular responses associated with vascular changes Hepato‐specific cells in radiation‐induced liver injury	Nano‐Hap Nb2C‐PVP 188‐b‐PCL CAPE‐incorporated HAsPBPE nanoparticles CNPs–AL–PEG	Reduce free radicals to alleviate radiation‐induced hepatotoxicity	[62, 79, 100, 148, 205]
Spleen	Disturbance of the antioxidant system	Nanomelanin Fullerenol C60(OH)_24_ nanoparticles (FNP)	The transcription levels of TNF‐α and IL‐2 in spleen were promoted, and the spleen fibrosis of irradiated mice was effectively alleviated; FNP exhibit high anti‐oxidative and anti‐inflammatory potential	[206, 207]
Lung	Free radical mediated early apoptosis, intermediate inflammation of pneumonia, and potential fibrosis Cellular oxidative stress Radioactive pulmonary fibrosis	MnSOD; Cur‐Dox/MPEG‐PCL FNP Nb2C‐PVP 188‐b‐PCL cerium oxide nanoparticles (CONPs)	Decreased tumor necrosis factor TNF‐α, TGF‐β, and interleukin IL‐1; inhibition of NF‐κB pathway; FNP exhibit high anti‐oxidative and anti‐inflammatory potential; reactive oxygen species (ROS) scavenging	[62, 117, 148, 207, 208, 209, 210]
Kidney	Glomerular changes, tubule interstitial damage Vascular injury and functional changes	Nano‐silymarin; selenium nanoparticles(Se‐NPs) 188‐b‐PCL	Eliminate ROS and regulate cell cycle; selenium is a cofactor of many enzymes that regulated antioxidant defense and the immune system	[62, 80, 211, 212]
Skin	Inflammatory response Oxidative stress	FGF‐2&LMWH/P; VEGF—Chitosan	FGF can enhance radioresistance; by protecting epidermal stem cells; vascular endothelial growth factor (VEGF) is also a cytokine with radiological protection	[213, 214]
Brain	Cellular and biochemical dysfunction Inflammatory reaction	DF‐1; TPP‐CeO_2_; PEG‐MeNPs; PtPdMo	Target mitochondria and remove ROS in mitochondria; anti‐inflammatory effects; neuroprotective	[75, 143, 215, 216]
Gastrointestinal tract	Changes of intestinal microflora in intestinal barrier function It destroys the integrity of the tight junctions between intestinal epithelial cells	HSV‐MnSOD; GDY‐ bovine serum albumin NPs; CNSI BSA‐GTP‐Chitosan nanoparticles	MnSOD gene can prevent and cure radioactive enteritis; radicals scavenging; TPs reduces can restore the redox status through Nrf2‐ERK pathway and reduce Bax expression	[217, 218]

### 5.3 Unsatisfactory research strategies

**FIGURE 6:**
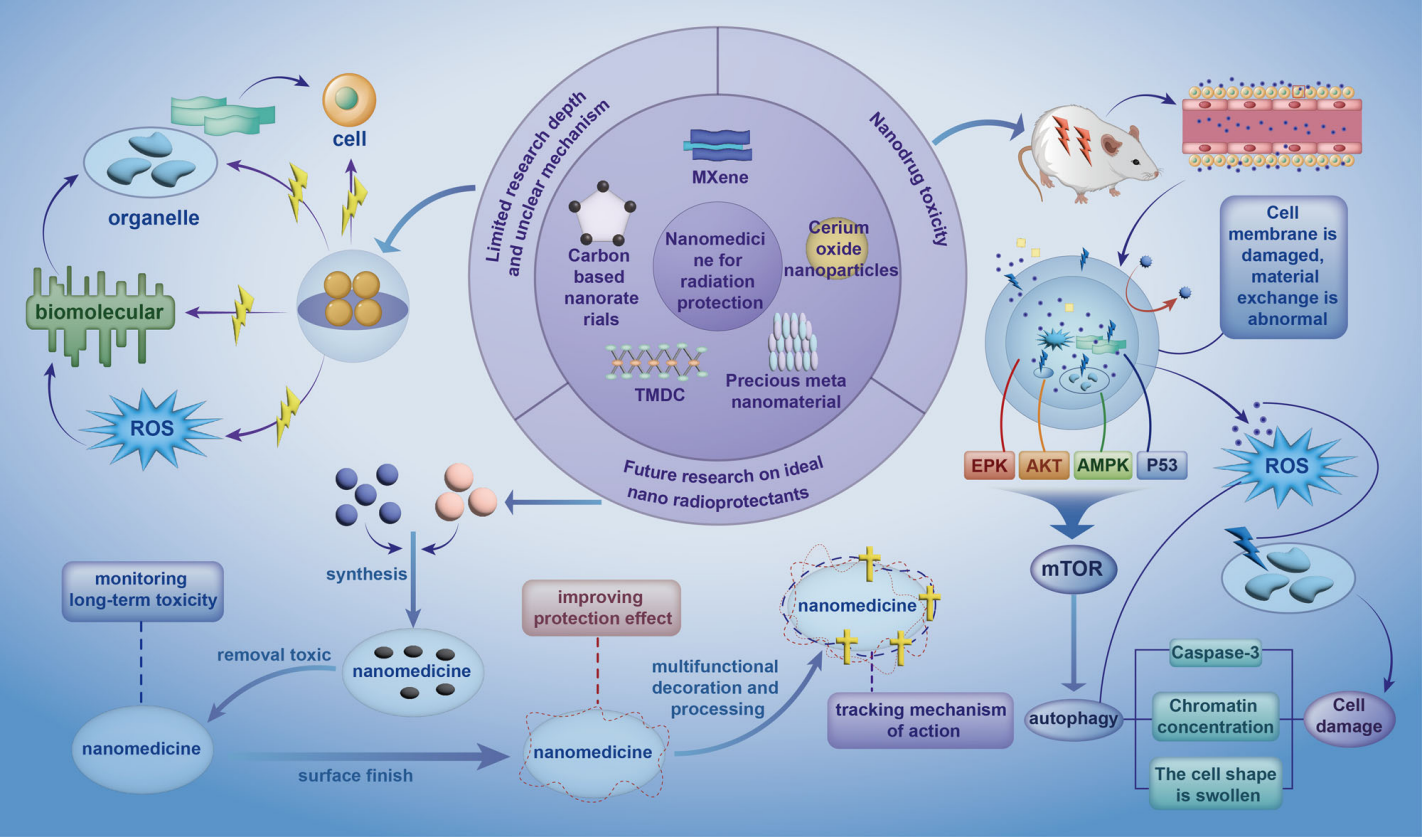
Limitations of previous studies of intrinsic radioprotective nanomaterials and recommendations for future research in this area. The radiation protection effect of nanomedicines can be enhanced by developing new nanomedicines or chemical modification. The molecular signal mediated by nanomedicines is deeply explored.

After going through the current mainstream research in this field thoroughly, a single mechanism as the entry point is usually adopted to tend to search for new drugs, which extensively served as the main research orientation, while neglecting the patience of continuous optimization and joint mechanism innovation around a promising class of key drugs. However, the latter is often regarded to be the key contributor to creating the most excellent radio‐protective drugs. The nanotechnology in the field of radioprotective drug development can bring many remarkable properties to the latter, but up to this day, researchers have mainly paid attention to only several properties such as antioxidant mimetic enzymes and nanocarriers to achieve the modification and optimization of protective drugs. However, the radiation damage process in biology is a multi‐level complex action involving subatomic, free radical, molecular, biomolecular, organelle, cellular, and even tissue levels, of which free radical and ROS stress are relatively upstream of the injury event chain, but not the only significant target. Therefore, the focus of the current research is relatively confined. Further systematic studies need to consider the chemical modifications, modification optimization, and molecular biosignal alterations of nanomedicines for combined mechanism innovation. In short, intensive development around key potential radioprotective candidates has become a growing tendency in this promising field (Figure 6).

## 6 OUR ADVICE FOR FUTURE RESEARCH IN THIS AREA

As discussed above, despite acquiring many promising developments in the application of nanotechnology to radiation protection medications, there is still a large gap to surmount to reach large‐scale practical applications. Based on the major reasons stated above, the following recommendations are listed for future studies.

First, future studies should concentrate on gaining a better understanding of both the radioprotective and toxic effects of newly‐designed nanodrugs. For example, the long‐term toxicity is strongly suggested to be monitored for a more convictive safety, with animal survival and organ function testing as some of the recommended assessments. If the toxicity is found to be so severe, the candidate should simply be aborted in general. For the most promising radioprotectors, however, but with significant toxicity, many factors including size effect, the composition of the nanodrugs, charge, surface reactivity, dose effect, targeted organs, and so forth should receive much attention and be fine‐tuned in detail to achieve a better applicable future chance.

Second, the research depth of nano‐radioprotective drugs needs to be systematically strengthened. To achieve a more objective overview, the in vitro experiments (such as cell culture testing), in silico methods (such as molecular docking, molecular dynamics simulations, and quantitative structure‐activity relationship modeling), ex vivo measurements (such as slice tissue culture, organoids, organ chips), in vivo testing (experimental animals) and clinical sample‐based means and even clinical trials should be comprehensively taken into account to evaluate the protective effects and toxicity. Meanwhile, the chosen test indices should be prudently arranged. For example, when performing the animal experiment, the pharmacokinetic assay and functional evaluations of different organs (such as renal function tests) should be adopted for drug safety evaluation.

Third, regarding the mechanism of nano‐drugs against radiation damage, the corresponding index effect based on the molecular drug design principle, especially for nano‐products with intrinsic anti‐radiation, should be carefully detected. As a powerful tool, approaches based on high‐throughput target screening such as proteomics and transcriptomics should also be incorporated to search for the specific molecular targets activated by nano‐radioprotectors. Next, the main targets screened should be deeply inspected by fully utilizing various molecular biology tools.

Last but not least, much more attention should be paid to the most potential nano‐radioprotectants by the invention of cooperative mechanisms and repeated reinforcement of performance. Researchers should make attempts to focus on applying favorable combinations to the same promising medication candidates based on a deep understanding of the physicochemical and biological mechanisms of nanomedicines to improve radioprotection. For one thing, collaborative innovation from multiple perspectives, such as preferential prototypical drug selection, carrier construction, dosage scheme design, and full biological signaling pathway utilization, should be performed to generate a refined product. For another thing, to generate strong and low‐toxicity radiation protectants, the joint optimization of different goals such as strengthening stability, improving bioavailability, lowering toxicity, and improving antioxidant capacity should be attempted. Therefore, it is strongly advised that researchers iteratively optimize and continuously improve the most promising research candidate to create high‐quality, practically‐useable nano radiation protection agents.

## 7 CONCLUSION

The development of nanomaterials dramatically increases the vitality of radiation protection, which plays a key role in preventing nuclear accidents, radiation exposure injuries, and so forth. Heretofore before this time, lots of studies in this field have been reported with much success. Rather than encompass other topics such as nano polymers delivering radioprotective drugs in the present review, we concentrate on the class of intrinsic nano‐radioprotectants to systematically discuss the recent developments in this area for the first time. In the first section, the history of radioprotective drugs and the difficulties related to their development are described. In the following section, the advantages and basic design principles of intrinsic nano‐radioprotectants are thoroughly explained. Next, the research advances of intrinsic radioprotective nanodrugs are analyzed with emphasis. Last, the limitations of the research are discussed along with several suggestions for future studies. Hence, we hope that this review will provide a comprehensive overview of intrinsic nano radioprotection and encourage further attention to this remarkably promising field.

## AUTHOR CONTRIBUTIONS

Jiaming Guo, Zhemeng Zhao, Huanhuan Zhu, and Kun Zhang constructed the skeleton and depicted the figures, Jiaming Guo, Zhemeng Zhao, Zeng‐Fu Shang, Zhongmin Tang, and Huanhuan Zhu wrote the manuscript, and Kun Zhang revised the manuscript. All authors commented on this manuscript and approved this submission.

## CONFLICT OF INTEREST STATEMENT

The authors declare no conflicts of interest.
